# Inguinal Hernia Incarceration in the Setting of Postoperative Ileus

**DOI:** 10.7759/cureus.35737

**Published:** 2023-03-03

**Authors:** Caline McCarthy, Whiyie Alfanso Sang, Mena Bekhit

**Affiliations:** 1 Department of Surgery, University of Central Florida College of Medicine, Orlando, USA; 2 Department of Colon and Rectal Surgery, Orlando Health, Orlando, USA; 3 Department of Surgery, Orlando Veterans Affairs Medical Center, Orlando, USA

**Keywords:** lower gi or colorectal surgery, postoperative small bowel obstruction, small bowel obstruction, incarcerated inguinal hernia, postoperative ileus

## Abstract

Postoperative ileus (POI) occurs after gastrointestinal and other intra-abdominal surgeries, and its incidence rate is reported to range between 10 and 30% following major abdominal surgery. Should ileus remain for several days or if symptoms worsen despite management, further investigation is warranted to consider other diagnoses such as small bowel obstruction (SBO), intra-abdominal abscess, or perforation. The etiology of postoperative obstructive symptoms can evolve during the postoperative course and many possible factors contribute to postoperative gastrointestinal dysfunction. Prolonged POI may be a risk factor for hernia incarceration. We describe the case of a 72-year-old male with a history of perforated diverticulitis and Hartmann procedure status post-colostomy takedown complicated by prolonged POI for six days. Clinical workup revealed incarcerated inguinal hernia, which was treated with urgent inguinal hernia repair. Follow-up revealed resolution of gastrointestinal dysfunction within 48 hours of hernia repair.

## Introduction

Prolonged postoperative ileus (POI) occurs after gastrointestinal and other types of surgery. POI is characterized by bowel dysfunction and decreased motility, resulting in the ineffective passage of intestinal contents. Symptomatically, patients may present with abdominal distension, decreased or absent bowel sounds, constipation, and inability to advance oral intake [1]. The exact mechanism and causes of POI are not fully understood [2] but are thought to be due to factors such as inflammation, inhibitory neural reflexes, and neurohumoral peptides that disrupt the normal coordinated propulsive motor activity of the gastrointestinal tract [3]. Some degree of POI is an expected consequence of abdominal surgery. “Normal” POI is recognized as the return of bowel function illustrated by the passage of flatus or stool and tolerance of an oral diet by postoperative day (POD) four [4]. Estimates of the incidence of prolonged POI vary widely in the literature, depending upon the type of surgery. In a study of 27,560 patients undergoing elective colon resection from 2012 to 2013, researchers found “prolonged postoperative ileus occurred in 12.7% of patients, and the rate was highest in patients with ileocolonic anastomosis (15%)” [5].

Evaluation of an ileus should exclude other diagnoses such as small bowel obstruction (SBO), intra-abdominal abscess, or perforation, making ileus a diagnosis of exclusion. Prolonged ileus, defined as signs and symptoms of ileus on POD four or after [4], continues to worsen despite management, and further investigation and imaging are warranted [1]. Early postoperative small bowel obstruction (EPSBO), defined as obstruction within the first 30 days after surgery, is a distinct clinical state that is often difficult to differentiate from POI [6], differentiated by the lack of a transition point on CT or the presence of colonic gas on radiographic imaging [7]. In 90% of cases, SBO is caused by adhesions, hernias, or neoplasms [8].

Incarcerated inguinal hernia in the setting of POI is rare, with only a handful of reported cases in the literature [9]. We report a case of a 72-year-old male with EPSBO due to incarcerated inguinal hernia occurring in the setting of POI.

## Case presentation

**Figure 1:**
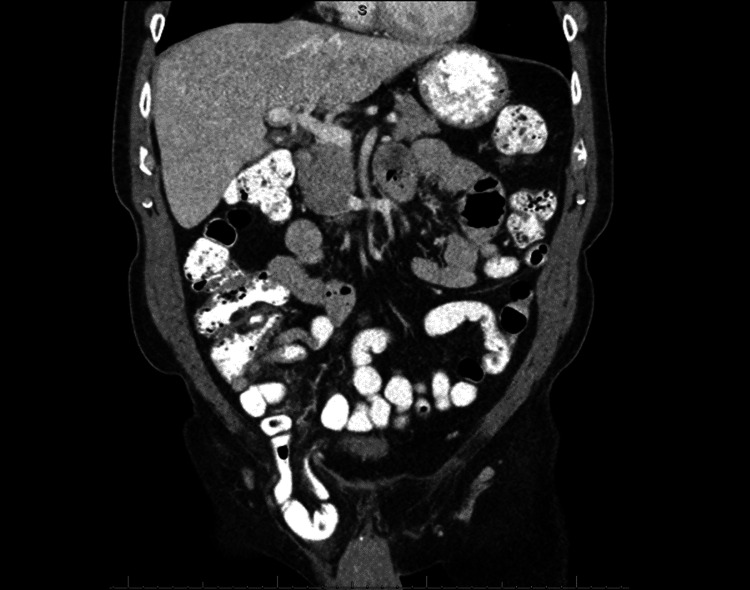
CT showing known right inguinal hernia with the loop of small bowel CT: computed tomography

A 72-year-old male presented to the colorectal team for Hartmann’s procedure reversal (colostomy takedown). He had a past medical history of perforated diverticulitis status post (s/p) open Hartmann procedure 12 months ago resulting in the current colostomy. Patient imaging on chart review revealed a six-month history of moderate-sized right inguinal hernia containing a loop of non-obstructed small bowel as seen in Figure 1.

Prior to the colostomy takedown, the patient had reported multiple, daily, soft stool outputs out of the colostomy bag. Before the colon resection in 2021, the patient’s bowel habits had included daily firm bowel movements with minimal straining. He denied any known history of hernias past or present. Past medical history was significant for diverticulitis, dyslipidemia, pulmonary emphysema, kidney stones, chronic rhinitis, and schizophrenia. Past surgical history was significant for colon resection for perforated diverticulitis with colostomy in 2021. Recent medication changes were limited to recent surgery and hospital admission. Family history was noncontributory. He had no known allergies.

**Figure 2:**
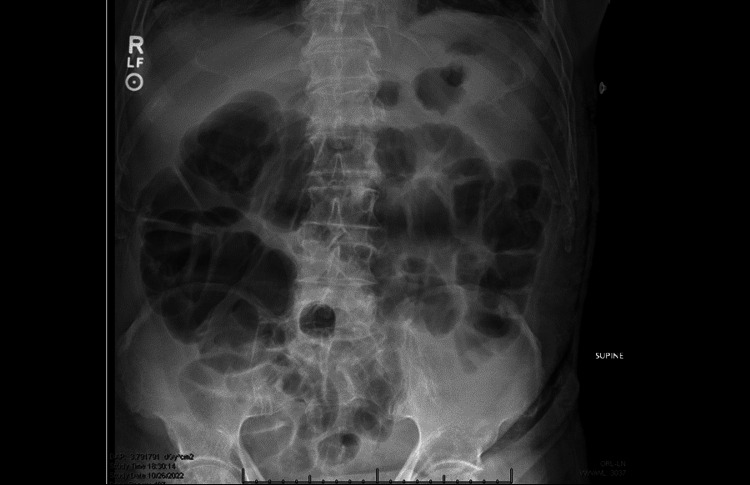
Abdominal X-ray from POD 1 showing ileus gas pattern with gaseous distention of several loops of the small and large bowel POD: postoperative day

Robotic-assisted laparoscopic reversal of Hartmann’s procedure and lysis of adhesions was performed. Intraoperative findings involved a right inguinal hernia with a loop of small bowel that was reduced during surgery. Postoperatively, the patient was advanced to clear liquids on POD zero. Oral intake caused significant nausea and vomiting, and the patient was made nothing by mouth (NPO) on POD one, which alleviated nausea. An abdominal X-ray was performed, which showed significant mild gaseous distention of several loops of the small and large bowel, nonspecific in the postoperative setting with no evidence for high-grade intestinal obstruction or large pneumoperitoneum as seen in Figure 2.

The patient continued passing flatus and burping but had no bowel movements. POD three through four, he was advanced to clear liquids and then a full liquid diet. On POD five, he had an irregular dark bowel movement thought to be from recent surgical anastomosis and was advanced to a low-fiber diet. On POD six, he developed nausea, and bilious vomiting, and continued to produce dark bloody stool with ongoing significant abdominal pain. His diet was changed back to NPO. The patient refused nasogastric (NG) tube insertion at this time.

Physical exam

The patient’s vitals on POD six s/p colostomy takedown were as follows: temperature of 36.9 °C, blood pressure of 138/75 mmHg, heart rate of 84 bpm, respiratory rate of 16, and pulse oximetry of 98% on room air. The physical exam showed a 72.57 kg male resting in bed in mild distress. The patient reported nausea and two episodes of emesis. He had bilaterally palpable carotid, radial, femoral, and posterior tibial pulses. Auscultation failed to appreciate any carotid bruits. Chest sounds revealed bilaterally clear breath sounds with no crackles, wheezes, or rales. His abdomen was mild to moderately distended and firm and tender to palpation in the left lower quadrant with normoactive bowel sounds. Inspection and palpation failed to appreciate any abdominal masses. A right inguinal hernia was appreciated upon visual inspection and palpation. The hernia was not able to be reduced. His abdomen revealed prior surgical incisions from the recent colostomy takedown.

Diagnostic studies/clinical decision-making

Recent blood tests showed no leukocytosis and no electrolyte abnormalities involving potassium, calcium, or magnesium. He was found to have a white blood cell count of 9.4 x 10^9^/L, hemoglobin of 14.2 mg/dL, platelet count of 322,000, and glucose of 153 mg/dL.

**Figure 3:**
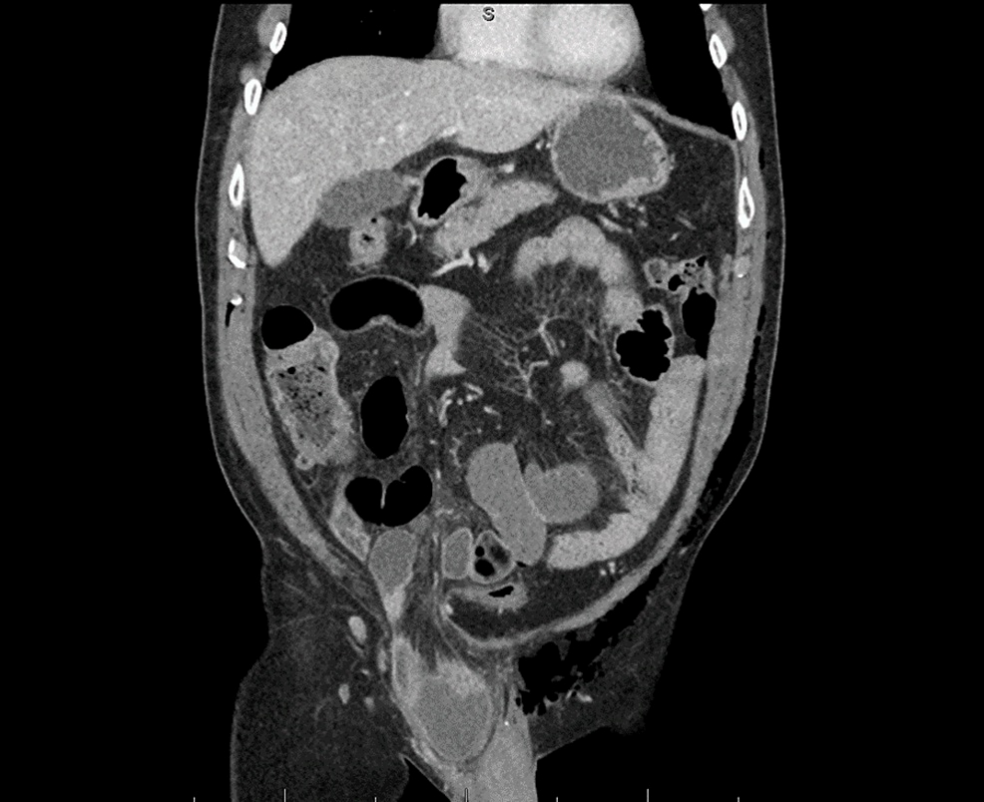
CT showing small bowel incarceration of the right inguinal hernia containing small bowel now with fat stranding and a dilated, fluid-filled loop of the small bowel, with associated upstream intra-abdominal small bowel dilatation and gas fluid levels, suggesting small bowel obstruction CT: computed tomography

CT imaging of the abdomen and pelvis with contrast was ordered to rule out an anastomotic leak, abscess, or SBO. Imaging findings suggested incarceration of a known right inguinal hernia containing small bowel, with associated upstream small bowel dilation and gas fluid levels, suggesting SBO as seen in Figure 3. CT imaging of the abdomen and pelvis with IV contrast five months prior to surgery had identified a right inguinal hernia recognized as moderate in size (Figure 1). The patient denied a history of any inguinal bulge, tenderness, or awareness of the hernia on prior imaging.

Several attempts were made to manually reduce the hernia, even by sedating the patient and positioning him in Trendelenburg. However, all attempts were unsuccessful. He had inguinal pain and tenderness on examination with persisting signs of SBO. Urgent operation for right inguinal hernia repair with relief of obstruction and possible small bowel resection was recommended with NG tube placement.

Treatment

The usual treatment approach for SBO caused by incarcerated hernia is urgent operation as it is significantly associated with risk for strangulation [10]. The patient was taken to the operating room for open right inguinal hernia repair. The hernia was repaired via a right inguinal incision with mesh enforcement and an NG tube was placed. Because the bowel was reduced with the induction of anesthesia, the bowel vascularity could not be assessed at the time of surgery. The patient was observed clinically post-op to ensure there was no evidence of bowel compromise.

Follow-up

On POD two, abdominal X-ray findings reflected improving SBO and the NG tube was removed. He was evaluated and found to have no nausea or vomiting, and a soft, non-tender, non-distended abdomen on physician exam. The patient’s diet was advanced. He had a return of bowel function, thereby demonstrating the resolution of bowel obstruction, and was discharged home.

## Discussion

We described a case of a 72-year-old male presenting with nausea, vomiting, diet intolerance, distended tender abdomen, and bloody stools in the postoperative setting of a colostomy takedown initially suspected to be prolonged POI. While this clinical picture can be explained by several possible pathologies, it was eventually found to be EPSBO due to incarcerated hernia.

EPSBO may present with an initial return of bowel function and initiation of oral intake with subsequent nausea, vomiting, abdominal distention, and pain, as in this case. Compared to this, a postoperative patient presenting with an ileus usually lacks the return of bowel function or advancement of oral intake [11]. Intense pain, feculent emesis, or rapid onset/progression of pain/distension are usually signs of obstruction and not of an ileus [11]. Management of POI relies on supportive care after excluding more serious and reversible surgical conditions like mechanical obstruction. Risk factors for prolonged POI after major abdominal surgery include, but are not limited to, male sex, respiratory comorbidities, operation lasting more than three hours, and peripheral vascular disease [12].

Explanations for the progression of this case involve two theories. The existing hernia may have presented with intermittent SBO and corresponding symptoms of abdominal distension, nausea, vomiting, and abdominal pain that may have mimicked symptoms of POI. Alternatively, symptoms from existing ileus, such as vomiting, may have escalated the hernia from asymptomatic to incarcerated. In the event of increased intra-abdominal pressure, hernia contents can be forced into the point of weakness in the abdominal wall and unable to be reduced [9]. Intra-abdominal pressure changes pose risk for hernia development. While more common causes of increased intra-abdominal pressure such as chronic cough, straining, and heavy lifting are typically discussed, vomiting has been documented as causing higher intragastric pressures than coughing or lifting [13]. Along with this theory, episodes of vomiting should be considered a significant risk factor for both hernia formation and existing hernia incarceration.

Management of asymptomatic hernias prior to major abdominal surgeries that pose a significant risk of POI may decrease the need for surgical interventions for postoperative hernia incarceration [14]. Considerable research exists surrounding the management of parastomal site hernia management in ostomy takedown procedures, as incisional site hernias develop in as many as 31-47% of temporary stoma sites [15,16]. While definitive management for symptomatic inguinal hernias is surgery [17], we were unable to identify guidelines to direct the management of asymptomatic inguinal hernias before major abdominal surgery. Nationally recognized guidelines suggest that “watchful waiting” in men of all ages with asymptomatic or minimally symptomatic inguinal hernias is a safe and appropriate early management strategy [18]. A scheduled elective hernia repair of an existing hernia, even if asymptomatic, prior to a major abdominal surgery, may significantly decrease the risk of pre-existing hernia intensification to incarceration and strangulation. 

An alternative approach is concurrent stoma closure and hernia repair compared to a staged procedure. In theory, this approach would reduce net recovery times and eliminate the need for two separate major abdominal surgeries compared to a single surgery. According to Oma et al., synchronous repair of the hernia and colostomy reversal led to a longer hospital stay and increased risk of reoperation for hernia recurrence compared with incisional hernia repair only [19]. Rudnicki et al. also found that the synchronous approach led to a higher complication rate compared to a staged hernia repair after the Hartmann reversal [20].

This case also highlights that many patients with inguinal hernias are asymptomatic, as this patient denied a history or current complaint of groin bulge or tenderness. A detailed history and physical exam supplemented by CT imaging are vital in identifying the site of obstruction. Imaging five months prior had documented his right inguinal hernia, which further proved to be significant as a means of comparison to current imaging and presentation. Incarcerated hernias pose a concern for strangulation, posing significant morbidity and adverse outcomes, and should be managed with emergency surgical repair [21]. Distinguishing between reversible ileus and mechanical obstruction at onset and through the clinical course is crucial as it significantly alters medical management and surgical intervention. Additionally, patients with prolonged gastrointestinal dysmotility are at greater risk of postoperative complications such as thromboembolic complications, intra-abdominal infections, delayed wound healing, and anastomosis leakage, which can lead to additional surgeries, longer hospital stays, and a higher risk of infection [22].

## Conclusions

Physicians should be cognizant that the etiology of postoperative obstruction can change during the postoperative course. As for this case, we theorize that the patient may have initially presented with uncomplicated POI due to inevitable pathophysiological factors related to abdominal surgery. However, the condition eventually evolved into mechanical obstruction, altering the course of management significantly. In most patients with prolonged POI, reliable baseline tests, and routine imaging are useful in narrowing down the differential and ruling out an underlying cause of mechanical obstruction.
